# Penny Wise and Pound Foolish

**Published:** 1911-03

**Authors:** 


### EDITORIAL DEPARTMENT.

### PENNY WISE AND POUND FOOLISH.

One of the chief obstacles in the way of any reform, or change in existing conditions, however evident and badly needed in the interest of race integrity, is indifference and ignorance on the part of those sent to Austin to make laws. It is almost impossible to awaken an interest in sanitation or hygiene; the lawmakers are so intent on—first, arranging for their pay and mileage, on adjournment just as soon as possible at the end of sixty days—when the five-dollar *per diem* ceases, and the two-dollar-a-day period begins,—and so intent is each one on getting passed some local hog law or fence law or some non-essential affecting only *his* constituency,—or on getting a statute against Sunday hunting, fishing or baseball playing, that such measures as the bill in the House by Dr. Parker of Robertson and Terrell of Wise in the Senate, providing for the arrest of procreation of degenerates,—*the biggest humanitarian movement of the century;* and the bill by Nickels, fathered by the Texas State Hygiene Society, prohibiting the marriage of the unfit, are side-tracked, and are so low on the calendar that they will not be reached in the short time of the regular term, most of which is taken up in unseemly squabbles on "rules," and in filibustering to prevent the passage of any liquor bills or redistricting bill; and should a called ($5) session be necessary, to enable them to pass the appropriation bill—the most essential feature of each Legislature—legislation would be restricted to that, or such measures as the Governor especially advises. True, the House has interested itself in the subject of tuberculosis, and two bills for State sanitaria are in motion there; but the conflict between them is apt to result in the defeat of both, while, at this writing, neither has reached the Senate. Action on this subject was secured only by the fact that the Governor, in fulfillment of his pledges to the medical profession, requested it. I should, therefore, qualify the sweeping statement above made. Indeed, individual members talked to are, almost without exception, in favor of the vasectomy bill published herewith, and of the anti-marriage bill referred to in our February number; and the former was reported favorably by the Public Health Committee and printed, while the marriage bill passed the

House. The trouble is in getting further action on them, and the prospects, at this writing, are that they will both die on the calendar.

POISONING THE STREAM AT ITS SOURCE.—Another subject of the greatest importance as affecting the race—for all time—is the unrestricted sale of poisonous and habit-forming patent medicines. Our Pure Food and Drugs Law is only a label law, and should be amended so as to prevent the sale of such stuff, for instance, as "Mrs. Winslow's Soothing Syrup" for infants. This stuff, as is well known, contains morphine, and, while the Texas law forbids the sale of morphine, except on physicians' prescription, anybody can buy this syrup of morphine, and poison his or her baby with it. The law only requires that it shall be printed on the label that the syrup contains morphine; but the mother with a fretful baby does not look at the label; she thinks that the advertisement in all the papers is gospel truth, and is deceived by its fraudulent and false statement, and thus hundreds of thousands of babies are poisoned by a drug, the use of which—extending over a period of two or more years of infancy—must and does affect the whole of after life, tending to produce neurasthenia, and insanity. I print here the advertisement which appears daily in nearly every newspaper, and, as there seems to be no way to head off and stop this dreadful decoy, I call upon every physician who reads this to warn mothers, everywhere, against such seductive and destructive decoy. Hundreds of thousands of babies are drugged with this poison every night for the first two years of their lives. It is a shame that such should be permitted; but what can be done about it? I might say the same about the habit-forming use of soda fountain drinks made from coca leaves—the much advertised and almost universally drunk Coca-Cola. I have had men tell me that drinking this stuff creates a craving for it, and they can not get to work until they have tanked up on it.

MOTHERS!

**Don't fail to procure Mrs. Winslow's Soothing Syrup for your children while cutting teeth. It soothes the child, softens the gums, allays all pain, cures wind colic, and is the best remedy for diarrhea. 25 cents a bottle.**

And, it should be added, locks the little one in a poisonous stupor—called sleep—and fixes a habit that, in after years, perhaps leads to lunacy—or other degeneracy, or suicide. These people—the advertisers—have the ear of the public, and pay enormously for it, and the public believes them. The medical profession has no way of answering them, and thus the evil goes on unchecked, and the generations that follow are handicapped by the unfitness of their ancestors. The patent medicine evil is one that cries aloud for reform, and is far-reaching in its destructive effects on the human family.

THE FOLLOWING BILL, written by the editor of the TEXAS MEDICAL JOURNAL, and referred to above, is now on the calendar of both houses; but the time for adjournment is so near that I have little hope that it will be reached. In addition to the one million dollars last year ($958,555) that the insane cost the State (Comptroller's figures), I call attention to the Governor's last message, in which he says that the buildings of the State eleemosynary institutions and the State penal institutions are so dilapidated that it will require an appropriation of $2,500,000 to repair them, that sum being just one-half of the State's estimated annual revenue from all sources; and that, in order to effect these needed repairs, it will be necessary to *double* or *treble the tax rate!* But, as stated, such bills must be sidetracked to make way for some hog law or wolf-scalp law or Sunday observance law. Are we civilized?

IN THE INTEREST OF RACE INTEGRITY.

House Bill No...... By Parker.

A BILL

TO BE ENTITLED

*An Act to prevent the procreation of lunatics, epileptics, imbeciles, syphilitics and other forms of degeneracy by the inmates of the several State asylums; to appoint two surgeons in each town where a State institution for the insane is located, who, with the Superintendent of such institution, shall constitute a Medical Board to determine on and take such steps as may be necessary to sterilize certain patients in the manner herein provided.*

WHEREAS, Insanity, epilepsy, imbecility and syphilis are by heredity transmitted to succeeding generations, in consequence of which those forms of degeneracy are increasing with startling rapidity and in excess of increase of population, carrying with it a corresponding increase of cost, which, for the insane in Texas in 1910, was, in round numbers, one million dollars; and

WHEREAS, In consequence of the crowded condition of the insane asylums at all times, in order to relieve the jails and almshouses of pauper insane and care for them in the asylums, the practice obtains of indefinitely furloughing the incurable or "improved" insane; and

WHEREAS, Sterilization of man may be effected by a trifling operation which neither mutilates nor unsexes a person; therefore,

*Be it enacted by the Legislature of the State of Texas:*

SECTION 1. The Superintendents of the several State hospitals for the insane and for epileptics in this State are hereby authorized and directed to appoint for each of said institutions, respectively, two skilled surgeons, who, in conjunction with the physician or surgeon in charge at each of said institutions, shall constitute a board, the duty of which shall be to examine such inmates of such institutions as are reported to them by the Superintendent to be persons by whom procreation would be inadvisable. Such board shall examine the physical and mental condition of such persons and their record and family history, so far as the same can be ascertained and if, in the judgment of a majority of said board, procreation by any such person would procure children with an inherited tendency to insanity, feeble-mindedness, idiocy, or imbecility, and there is no probability that the condition of any such person so examined will improve to such an extent as to render procreation by any such person advisable, then said board shall appoint one of its members to perform the operation of vasectomy upon such person, prior to furlough or discharge. Such operation shall be performed in a safe and humane manner, and the surgeon performing such operation shall receive from the State the sum of $3.00 as compensation for such operation performed.

---

SHOULD ANY ONE SAY that I have been unjust in the charge above made, let him read the *San Antonio Express* of February 23d (ult.). The staff correspondent quotes the Governor as saying that "if the Legislature fritters away the rest of the sixty days of the session, as most of the time since January 10th has been wasted, it will not be my fault." This raised a tremendous row, and in a heated wrangle over a resolution censuring the Governor,—amongst other very pointed remarks, Mr. Nickels of Hill said, "Isn't it a fact that the newspapers have devoted several columns daily to the Senate, where they have been *doing* nothing but squabbling?"

I repeat, things worth while have been neglected and what little legislation has been effected—with little exceptions—has been unimportant.

THE GREATEST ABSURDITY of the age, next to licensing osteopaths (without examination on materia medica, therapeutics and principles of practice) to practice medicine and surgery in all its branches, is what is called the "Optometry Bill." The House Committee on Public Health reported it favorably on February 25th, despite the protest of the State Health Officer, and, practically, the entire State Medical Association. But—it will not be reached.

